# Pandora’s box

**DOI:** 10.1192/bji.2025.10049

**Published:** 2025-08

## On global politics, economics and health

The changing political climate, with major socioeconomic consequences affecting the most vulnerable, has major health implications around the globe. As civilian mortality in conflict zones is rising shockingly, health provision in both ‘war and peace’ is rapidly declining, particularly for those most in need. The withdrawal of aid to low-income countries by the rich and the slashing of financial support to the World Health Organization (WHO) by the USA have significantly worsened the situation, not long after the pandemic. Dr Tedros Adhanom Ghebreyesus, Director-General of the WHO, despairs: ‘How can WHO be expected to serve the whole world on the same budget as one hospital in a mid-sized European city’. The WHO has had to reduce its workforce, as well as the scale and scope of its work and adjust the targets of the Fourteenth General Programme of Work, limiting its focus to core functions.

A glimpse of hope appeared after a meeting of the World Health Assembly (WHA), where member states reaffirmed their commitment to the WHO and demonstrated multilateral action to protect and promote public health. The Director-General stated: ‘the World Health Assembly has sent a clear message: countries want a strong WHO and are committed to working together with WHO to build a healthier, safer and fairer world. These were strong votes of confidence in WHO at this critical time’. The WHA adopted the pandemic agreement on 20 May this year, a landmark in the history of WHO and global health. There was also approval of the WHO’s 2026–2027 programme budget, with a rise in contributions agreed by member states, increasing these progressively to 50% of the base budget. This is seen as a major move towards making the WHO more independent and less reliant on voluntary funds from a small number of traditional donors. Several important resolutions were also made, including a new target to halve the health impact of air pollution by 2040. Other resolutions adopted by the WHA included attention to digital health, sensory impairment, social connection and several other areas concerning global health.

World Health Organization (WHO). WHO Director-General’s Opening Remarks at the 42nd Meeting of the Programme, Budget and Administration Committee of the Executive Board – 14 May 2025. WHO, 2025.

World Health Organization (WHO). *WHO Director-General: Member States Reaffirm Commitment to WHO and Global Health at Historic World Health Assembly*. WHO, 2025.

## Genes and health for all – the Brazilian example

Brazil is among the most ethnically mixed and diverse countries in the world, and its population of 200 million has seen a large reduction in numbers of the indigenous people over the years, through disease, displacement and warfare, the forced arrival of 5 million Africans during the slave trade in the 16th to 19th century, and colonisation by about 5 million Europeans; to these changes are added the arrival of more recent immigrants from Asia and the Middle East. This genetic diversity has been demonstrated in a massive genomic study, which also identified a large number of new variants related to disease. This multimillion-dollar project funded by the country’s Ministries of Health and Science is intended to sequence the whole genome of 100 000 Brazilians before the year 2030, with the aim of improving health via advances in precision medicine. Existing genetic databases are biased towards European genetic variations, and the researchers in this study are ensuring the inclusion of the indigenous populations in the region of the Amazon.

The colonial past of Brazil is reflected in their findings so far, with about 59% of Brazilians having European ancestry, 27% African ancestry and only 13% indigenous American ancestry. The Brazilian population differs from that of other Latin American countries such as Mexico. In one study in Mexico City, the indigenous population was estimated to be 66%, compared with 31% for European and 3% for African ancestry.

Notably, 71% of Y chromosomes (transmitted by males) came from those of European ancestry, whereas mitochondrial DNA (transmitted by females) was 42% for those with African and 35% for those with indigenous heritage. The researchers attribute these findings to sexual coercion of women of indigenous and African origin by European men during the colonial period.

Very importantly for health, the study has identified more than 8.7 million genetic variants that were not previously known and are unique to Brazil, with 36 000 of these considered to be potentially damaging to health and having roles in various disorders including hypercholesterolaemia, obesity, malaria and tuberculosis. It is hoped that these results will help efforts to develop disease prevention strategies and, in particular, improve the health of marginalised communities. An example is the genetic variant from those of African origin that protects against the sleeping sickness caused by the parasite *Trypanosoma brucei*, which is also associated with an increased risk of chronic kidney disease in Black Brazilians.

Hopefully this Brazilian initiative will serve as an example to others and encourage state spending on the health and well-being of populations rather than for military purposes.

**Pérez Ortega R.** Massive DNA sequencing effort reveals how colonization shaped Brazil’s genetic diversity. *Science* 2025. Available from: https://doi.org/10.1126/science.zft36gr.

## How humane are we humans?

The current world situation doesn’t inspire much confidence in our common humanity. Nevertheless, we human beings consider ourselves to be social animals, and our daily personal and work lives involve social interactions. How successful we are in social interactions is thought to influence our ability to climb up the social and professional ladder. This is not in keeping with research showing that people from lower social status are better at perceiving other people’s emotions, which would be expected to make them more successful in social interactions. The question, therefore, is whether the ability to perceive other people’s emotions, called empathic accuracy, is indeed what shapes social status, or whether it is the other way round. In other words, does lower social status increase emotion perception, or does the presence of emotion perception facilitate advancement to higher social status?

Researchers in a recent study aimed to test this question in two ways: (a) they collected subjective and objective measures of social status and examined their predictive power with respect to emotion perception ability while controlling for age, gender, race, ethnicity, political affiliation, agreeableness and sense of power; and (b) participants were asked to self-report on current and childhood social status, to enable the researchers to separate effects associated with change in social status from those associated with current social status.

Their results showed a negative relationship between individuals’ social status and empathic accuracy (perception of others’ emotions). Those with lower social status were better at this. They also considered changes in social status across the lifespan and observed a negative relationship between this change in social status and emotion perception. The authors recognise the limitations of their findings, including small numbers and effect sizes and the need for further work on the subject. Nevertheless, these results do appear to reflect current trends in society – not surprisingly, perhaps – that the higher we stand in social status the less empathic we become towards our fellow humans.

**Lee VK, Khaw MW, Kranton RE, Huettel SA.** Higher self-assessed subjective social status is associated with worse perception of others’ emotions. *Sci Rep* 2025; **15**: 17188.

## Digital technology: friend or foe?

As our professional and personal lives get taken over by digital technology, we can’t help but wonder whether this is beneficial or harmful to our cognitive ability. Is the use of digital technology making us more vulnerable or more resilient to dementia as we grow older? A recent meta-analysis and systematic review examined whether lifetime exposure to technology impairs our cognition or promotes it.

The authors examined suitable studies published in MEDLINE, PsycINFO, CINAHL, ScienceDirect, Scopus, the Cochrane Library, ProQuest and Web of Science. These included observational or cohort studies that focused on general digital technology used by adults over the age of 50 years and included outcomes of either cognitive ability or a dementia diagnosis. They identified 136 papers that met the inclusion criteria, 57 of which were compatible with odds ratio or hazard ratio meta-analysis; the studies included 411 430 adults, of whom 53% were women

Reassuringly, they found that digital technologies were associated with reduced risk of cognitive impairment and also reduced time-dependent rates of cognitive decline. These results remained unchanged when taking into account demographic, social, economic, health and cognitive reserve factors. Nevertheless the authors recognised that further work was needed on the subject. For the time being, feel free to carry on using the technology without undue worry.

**Benge JF, Scullin MK.** A meta-analysis of technology use and cognitive ageing. *Nat Hum Behav* [Epub ahead of print] 15 Apr 2025. Available from: https://doi.org/10.1038/s41562-025-02159-9.

## Why do we conform?

Since living in societies, we have created rules that the majority of us obey. These include laws and regulations imposed by authorities but also general standards of behaviour in our personal, professional and political lives. What drives our conformity is not well understood: is it because we fear the consequences of non-conformity, because of a sense of duty, to meet social expectations, or because we consider the effects of our behaviour on others? Social scientists looked at this question using an interdisciplinary framework which they called CRISP – an acronym for explaining rule conformity (C), as a function of intrinsic respect for rules (R), extrinsic incentives (I), social expectations (S) and social preferences (P) – in a series of online experiments.

Reassuringly, they found that respect for rules and social expectations were the basic elements of conformity; this explains why we generally follow rules and social norms, even when there are no extrinsic incentives or social preferences. There is still some hope for humanity.

**Gächter S, Molleman L, Nosenzo D.** Why people follow rules. *Nat Hum Behav* [Epub ahead of print] 26 May 2025. Available from: https://doi.org/10.1038/s41562-025-02196-4.

